# Long Chain Fatty Acid Degradation Coupled to Biological Sulfidogenesis: A Prospect for Enhanced Metal Recovery

**DOI:** 10.3389/fbioe.2020.550253

**Published:** 2020-10-23

**Authors:** Anna Patrícya Florentino, Rachel Biancalana Costa, Yuansheng Hu, Vincent O'Flaherty, Piet N. L. Lens

**Affiliations:** ^1^Department of Microbiology, School of Natural Sciences and Ryan Institute, National University of Ireland Galway, Galway, Ireland; ^2^Department of Biochemistry and Organic Chemistry, Institute of Chemistry, São Paulo State University, Araraquara, Brazil; ^3^Department of Civil Engineering, School of Engineering, College of Science and Engineering, National University of Ireland Galway, Galway, Ireland

**Keywords:** sulfidogenesis, long chain fatty acid, beta-oxidation, metal precipitation, oleate toxicity

## Abstract

This research assessed the microbiological suitability of oleate degradation coupled to sulfidogenesis by enriching communities from anaerobic sludge treating dairy products with S^0^, ${\text{SO}}_{3}^{2-}$, ${\text{SO}}_{4}^{2-}$, and S_2_${\text{O}}_{3}^{2-}$ as electron acceptors. The limiting factor hampering highly efficient oleate degradation was investigated in batch reactors. The best sulfidogenic performance coupled to specialization of the enriched bacterial community was obtained for S^0^- and S_2_${\text{O}}_{3}^{2-}$-reducing enrichments, with 15.6 (± 0.2) and 9.0 (± 0.0) mM of sulfide production, respectively. Microbial community analyses revealed predominance of *Enterobacteraceae* (50.6 ± 5.7%), *Sulfurospirillum* (23.1 ± 0.1%), *Bacteroides* (7.5 ± 1.5%) and *Seleniivibrio* (6.9 ± 1.1%) in S^0^-reducing cultures. In S_2_${\text{O}}_{3}^{2-}$-reducing enrichments, the genus *Desulfurella* predominated (49.2 ± 1.2%), followed by the *Enterobacterales* order (20.9 ± 2.3%). S^0^-reducing cultures were not affected by oleate concentrations up to 5 mM, while S_2_${\text{O}}_{3}^{2-}$-reducing cultures could degrade oleate in concentrations up to 10 mM, with no significant impact on sulfidogenesis. In sequencing batch reactors operated with sulfide stripping, the S^0^-reducing enrichment produced 145.8 mM sulfide, precipitating Zn as ZnS in a separate tank. The S_2_${\text{O}}_{3}^{2-}$ fed bioreactor only produced 23.4 mM of sulfide precipitated as ZnS. The lower sulfide production likely happened due to sulfite toxicity, an intermediate of thiosulfate reduction. Therefore, elemental sulfur reduction represents an excellent alternative to the currently adopted approaches for LCFA degradation. To the best of our knowledge, this is the first report of oleate degradation with the flux of electrons totally diverted toward sulfide production for metal precipitation, showing great efficiency of LCFA degradation coupled to high levels of metals precipitated as metal sulfide.

## Introduction

Anaerobic reactors have been widely used for the treatment of lipid-rich wastewater, and, due to the high energy content of lipids, they are generally coupled to the production of biogas (Alves et al., 2009; Dasa et al., 2016). However, when no suitable feeding strategy is adopted and the accumulation of long-chain fatty acids (LCFAs) is not controlled, the efficiency of lipid-fed anaerobic digesters has been shown unpredictable (Hawkes et al., 1995; Hwu et al., 1998; Alves et al., 2009).

The release of LCFAs from fat, oil and grease (FOG)-rich wastewaters occurs through the activity of extracellular lipases that hydrolyze lipids into LCFA (Palatsi et al., 2009). Oleate (C18:1), stearate (C18:0), and palmitate (C16:0) are LCFAs commonly present in anaerobic digesters fed with lipids, from which oleate shows the highest abundance in wastewater systems (Komatsu et al., 1991), reasonably good solubility and high toxicity to anaerobic digestion communities (Lalman and Bagley, 2002; Zhang et al., 2011). Proton-reducing acetogenic bacteria present in the system degrade such fatty acids by performing the cyclic β-oxidation pathway, in which a recurrent cleavage of 2-carbon fragments occurs with concomitant release of acetyl-CoA (Nelson and Cox, 2005).

For biogas production, a syntrophic association of LCFA-degrading bacteria with hydrogen-utilizing microorganisms is required to guarantee a low partial pressure of hydrogen in the system (Sousa et al., 2007). Furthermore, when major products of β-oxidation accumulate to thermodynamically-limiting levels, further oxidation of LCFA and propionate is hampered, inhibiting the digestion to proceed (Labatut et al., 2014). In this scenario, the presence of LCFA has been reported to impact the activity of hydrolytic, acidogenic, acetogenic bacteria, and methanogenic archaea (Lalman and Bagley, 2002; Pereira et al., 2005). The archaeal community, however, is more resilient to increased LCFA concentrations compared to the bacterial community (Lalman and Bagley, 2002; Zhang et al., 2011; Ma et al., 2015), with partial inhibition of archaeal activity due to a reversible mineralization of LCFA (Pereira et al., 2005). Despite the reversibility of inhibition, the biogas production from the degradation of lipidic matter has yet several trammels on its way.

The difficulties related to LCFA-rich wastewaters for methane production raise the question whether other bioprocesses could be more suitable for handling this type of effluent. Sulfur cycle microorganisms, for example, are possible syntrophic partners, as they can generally use hydrogen as electron donor (Sánchez-Andrea et al., 2014; Florentino et al., 2016b). Besides, several microorganisms able to reduce sulfurous compounds are also able to directly oxidize LCFAs, without the requirement of syntrophic relationships for the complete degradation of these substrates (Bonch-Osmolovskaya et al., 1990; Miroshnichenko et al., 1999; Florentino et al., 2016a). In this case, the final product is hydrogen sulfide. The versatility of sulfidogenic microorganisms allows for many combinations of electron donors and sulfur sources, and for a wide range of operational conditions for the process.

The theoretical high levels of sulfide produced from the degradation of LCFAs can be utilized for the precipitation of heavy metals in solution, which is of great relevance from the biotechnological point of view, considering that the release of heavy metals in the environment is a major environmental problem (Akcil and Koldas, 2006; Moodley et al., 2017). Once sulfide is released, it can bind to divalent heavy metals in solution, such as Cu^2+^, Zn^2+^, and Ni^2+^, precipitating as insoluble metal sulfides, consequently generating a metal-free effluent. Therefore, it represents not only a strategy to alleviate metal-related pollution, but it also avails metal recycling (Johnson, 2014; Işildar et al., 2019).

In this study, we aimed to assess the microbiological suitability of LCFA degradation coupled to sulfidogenic bioconversions by enriching communities from anaerobic sludge treating dairy products. The performance of the enrichments was analyzed using different electron acceptors, and their tolerance to increasing concentrations of LCFA was studied. The limiting factors hampering efficient LCFA degradation were assessed. To the best of our knowledge, this is the first report of LCFA degradation with the flow of electrons totally diverted toward sulfide production for metal precipitation, with great efficiency of degradation yielding high levels of metal sulfides.

## Materials and Methods

### Inoculum Source, Media, and Screening Setup

Anaerobic sludge from a dairy industry in Mitchelstown (Co. Cork, Ireland) was used as source of inoculum. An aliquot (1 mL) of the homogenized sludge was added to 120 mL serum bottles with 50 mL sterile anoxic basal medium, prepared as described elsewhere (Stams et al., 1993). Briefly, the composition of the medium (g L^−1^) was: 0.41 KH_2_PO_4_; 0.53 Na_2_HPO_4_·2H_2_O; 0.3 NH_4_Cl; 0.3 NaCl; 0.1 MgCl_2_·6H_2_O; 0.11 CaCl_2_·2H_2_O; and 1 mL L^−1^ of acid and alkaline trace elements solution; 0.2 mL L^−1^ vitamins; 0.1 g L^−1^ yeast extract (Alfa Aesar, Ward Hill, MA) and 1 mL L^−1^ resazurin sodium salt solution (Fischer Scientific, Hampton, NH). The initial pH of the medium was set to 6.8 by addition of HCl or NaOH. Bicarbonate-buffer was not added to the medium, in order to monitor the pH values over time and to understand the impact of the LCFA degradation on the pH in the enrichments. Serum bottles were sealed with butyl rubber stoppers (Ochs Laborbedarf, Bovenden, Germany) and flushed with a 1.5 atm N_2_ headspace. Enrichments were incubated statically in the dark at 30°C.

Supplementary Figure 1 shows the experimental procedure flow chart. Four sets of transfers were performed to assess the oleate degradation coupled to sulfidogenic activity with elemental sulfur (S^0^), sulfite (${\text{SO}}_{3}^{2-}$), sulfate (${\text{SO}}_{4}^{2-}$), and thiosulfate (S_2_${\text{O}}_{3}^{2-}$) as electron acceptors. The mentioned compounds were added to the enrichments to a final concentration of 25 mM. Sodium oleate 82% (Sigma-Aldrich, St. Louis, MI) was added as electron donor and carbon source from sterile anaerobic stock solutions to a final concentration of 1 mM. Two series of controls were performed without any external electron donors or electron acceptors. Activity was monitored every 4 days by sulfide production, volatile fatty acids and hydrogen profile, and pH dynamics. Screening cultivation experiments were performed in triplicates.

Four sets of serial dilutions were performed (Supplementary Figure 1). The serial dilutions were performed to eliminate the influence of organic matter present in the sludge, which would play the role of electron donor instead of the added LCFA. After the 4th transfer, as the endogenous organic matter was completely depleted in the cultures (no peaks or organic acids or sugars chromatographically detected), the sulfide production could thus be correlated solely to LCFA degradation. Results in this manuscript report the data of the 4th transfer only.

### Sequencing Batch Reactors

Screening enrichments presenting the best LCFA-degrading performance and with the highest selective sulfur-metabolizing microbial community after the 4th transfer were selected for further analyses in a sequencing batch reactor (SBR). Selected enrichments were used as inoculum source (10%, v/v) in 1020 mL glass-made SBR, with 500 mL working-volume and 520 mL headspace. Bioreactors were flushed with N_2_ for 15 min and the final pressure was set at 1.5 atm N_2_ to ensure an O_2_-free headspace. The SBR were maintained at 30°C with the aid of thermostatic recirculation bath and submitted to an intermittent upflow recirculation flow rate of 46.5 mL min^−1^, and upflow velocity of 0.05 cm min^−1^ for 15 min every 6 h. The SBR were supplied with elemental sulfur (in excess) and thiosulfate (50 mM) as electron acceptors, and 2 mM sodium oleate 82% (Sigma-Aldrich, St. Louis, MI) as electron donor and carbon source. Activity of the biomass was monitored every 4 days by sulfide production, volatile fatty acids (VFAs) and pH dynamics.

A batch cycle consisted of 35 days for the thiosulfate-reducing reactor and 39 days for the sulfur-reducing reactor. At the end of each cycle, 20% (v/v) of the medium fed with substrates were replenished to avoid limitation of substrates or other nutrients. To prevent massive biomass loss, medium replenishment was always performed 5.5 h after the last recirculation cycle.

To release the potential negative impact of sulfide accumulation in the system, hydrogen sulfide was stripped every 9 and 17 days for, respectively, thiosulfate- and elemental sulfur-fed SBR. Hydrogen sulfide stripping was performed by flushing the SBR with N_2_ for 5 min (200 mL N_2_ min^−1^). N_2_ was flushed through a porous stone installed at the bottom of the SBR to ensure smooth mass transfer. At the end of the flushing procedure, the reactor headspace was put under 1.5 atm pressure. The hydrogen sulfide stripped from the bioreactors can be used for the precipitation of Zn^2+^ as ZnS. To assess metal precipitation with sulfide generated by the SBR, the gas stripped from the reactor was directed to a vessel containing 100 mL of a 5% ZnCl_2_ solution (w/v). The ZnS precipitates were left settling overnight. Zinc and sulfide concentrations in the supernatant and in the precipitates were then determined.

### Sacrificial Assays

To assess the profile of oleate degradation in S^0^ and S_2_${\text{O}}_{3}^{2-}$ -fed biomass, 56 serum bottles (30 mL) were filled with the above-described anoxic basal medium and inoculated with the enriched biomass (10%, v/v) present in the reactors sampled on the 50th day of operation. Triplicate bottles were sacrificed twice a week and the whole content of the bottles was used for pH, sulfide, VFA, and LCFA measurements.

### Oleate Tolerance Tests

To determine the inhibitory oleate concentration to the enriched microorganisms, elemental sulfur- and thiosulfate-reducing enrichments (using the 4th transfer biomass inoculated to the bioreactors) were incubated with sodium oleate 82% (Sigma-Aldrich, St. Louis, MI) in a concentration range from 1 to 10 mM, exceeding the reported inhibitory concentrations for anaerobic microorganisms (Hwu and Lettinga, 1997; Sousa et al., 2013; Silva et al., 2016). Experiments were conducted in triplicates and the bottles were incubated statically at 30°C. Activity was monitored every 4 days by sulfide production and pH dynamics.

### Sulfite Tolerance Tests

To investigate the impact of sulfite on the thiosulfate-reducing SBR biomass, incubations with initial concentrations of sodium sulfite of 2.5, 5, 7.5, 10, and 25 mM were performed with 1 mM sodium oleate 82% (Sigma-Aldrich, St. Louis, MI) added as electron donor and carbon source. A bioreactor sample from the 58th day of operation was used as inoculum. Experiments were conducted in triplicates and cultures were incubated statically at 30°C. Activity was monitored every 4 days by sulfide production and pH dynamics.

### Transmission Electron Microscopy (TEM)

Reactors were sampled on day 50th for TEM analysis. Fixation and imaging of cells were performed at the Center for Microscopy and Imaging at the National University of Ireland Galway. To cross-link cellular components and to preserve and stabilize cellular structures, samples were fixed in 2% (v/v) glutaraldehyde and 2% (w/v) paraformaldehyde in 0.1 M sodium cacodylate buffer/HCl pH 7.2 for 2 h, followed by fixation in 1% (w/v) osmium tetroxide in a 0.1 M cacodylate buffer/HCl (pH 7.2) for 2 h. Osmium-fixed samples were dehydrated in graded alcohol solutions, and pure ethanol was washed out by acetone replacement. Dehydrated samples were embedded in Agar Low Viscosity Resin (Agar Scientific, Stansted, UK). Complete polymerization of the resin occurred in an oven at 65°C for 48 h. Survey sections of 500 nm were cut using a glass knife and transferred to a glass slide, stained with toluidine blue and viewed using a light microscope. When a region of interest was identified, ultra-thin sections (70–90 nm) were cut with a diamond knife, filled on copper grids, stained with uranyl acetate and lead citrate to enhance organic matter contrast on a Leica EM AC20 automatic contrasting system (Leica, Wetzlar, DE), and examined in a Hitachi H7000 transmission electron microscope (Hitachi, Tokyo, JA).

### DNA Extraction

Cultures enriched in the screening were considered for microbial community analyses. Cells were harvested at the early stationary phase by centrifuging samples at 10,000 × g for 5 min. Triplicate harvested cells were used for DNA extraction with the DNeasy PowerSoil Kit (QIAGEN, Hilden, Germany), following the instructions of the manufacturer. Purity and concentration of the extracted DNA were checked in a NanoDrop 2000C (ThermoFisher, Scientific Walthan, MA). DNA concentrations were about 25 ng μL^−1^, with 260/280 ratios of ~1.8. Extracted DNA samples were stored at −20°C until sequencing. No-template-controls and extraction blanks were performed and resulted in no amplification when universal primers for bacteria and archaea were employed.

### Sequencing and Microbial Community Analysis

Frozen DNA samples were dispatched to Eurofins Genomics (Germany), where PCR was performed followed by sequencing on the Illumina MiSeq platform. Bacterial 16S rDNA genes were amplified using bacterial universal primers sequencing the hypervariable regions V3-V4 of the gene. Once the amplicons were obtained, all the reads with ambiguous bases (“N”) were removed, and chimeric reads were identified and removed following the UCHIME algorithm (Edgar et al., 2011) as implemented in the VSEARCH package for sequence analysis (Rognes et al., 2016). The remaining set of high-quality reads, the operational taxonomic unit (OTU)-picking strategy, was processed using minimum entropy decomposition—MED (Eren et al., 2013, 2015). The MED procedure identified and filtered sequences with a very low abundance (< ≈ 0.02% of the average sample size). DC-MEGABLAST alignments of cluster representative sequences to the sequence database (NCBI_nt—release 2019-01-05) were performed to assign taxonomic information to each OTU. The most specific taxonomic assignment for each OTU was then transferred from the set of best-matching reference sequences. The minimum requirement for a reference sequence was an identity of 70% across at least 80% of the representative sequence. Further processing of OTUs and taxonomic assignments was performed using the QIIME software package (version 1.9.1, http://qiime.org/). Abundances of bacterial taxonomic units were normalized using lineage-specific copy numbers of the relevant marker genes to improve estimates (Angly et al., 2014). The processed Illumina Miseq reads were deposited in the Sequence Read Archive of NCBI under accession number PRJNA622591.

### LCFA Extraction

Extraction and transmethylation of LCFAs were performed as previously described by Guihéneuf et al. (2014). Briefly, freeze-dried samples - about 10 mg dry weight (Lyovapor L-200, Buchi Labortechnik AG, Flawil, CH) received 2 mL of 2% H_2_SO_4_ in dry methanol in 15 mL glass vials with teflon sealed caps. 10 μL of the internal standard, pentadecanoic acid (5 mg L^−1^), were added to the solution under N_2_ flow, and heated to 80°C for 1.5 hour with continuous stirring. When at room temperature, 1 mL of H_2_O was added to terminate the transmethylation reaction, followed by the addition of 1 mL hexane to extract the fatty acid methyl esters (FAMEs), concentrated in the upper layer.

### Analytical Methods

The partial pressures of hydrogen and methane were measured in an Agilent 7890A gas chromatograph (Agilent Technologies, Santa Clara, CA), equipped with a 13803-U stainless-steel column (Sigma-Aldrich, St. Louis, MI) and a thermal conductivity detector. Temperatures of the oven and detector were set to 90 and 200°C, respectively. Argon was used as carrier gas at a flow rate of 24 mL min^−1^. VFAs were quantified using a 1260 Infinity II Liquid Chromatograph system (Agilent Technologies, Santa Clara, CA), equipped with a Hi-plex H column, a refractive index detector, and 14 mM H_2_SO_4_ eluent at a flow rate of 0.4 mL min^−1^.

Sulfide concentration in solution was determined by the photometric method using methylene blue as described previously by Cline (1969), after preservation of the sample by addition of 5% ZnCl_2_ solution (w/v). Sulfate, sulfite and thiosulfate concentrations were quantified using a Dionex Aquion ion chromatograph equipped with an IonPac AS14A 4 × 250 mm column, an AG14A 4 × 50 mm guard column, and a suppressed conductivity detector (ThermoFisher Scientific, Waltham, MA). The eluent was prepared as a mixture of carbonate and bicarbonate solutions to a final ratio of 3.03 mM / 0.97 mM, at a flow rate of 1 mL min^−1^.

LCFA concentrations were quantified as FAMEs, using an Agilent 7890A GC equipped with a flame ionization detector and a fused silica capillary column DB-WAXETR (Agilent Technologies, Santa Clara, CA). The measuring conditions followed the protocol proposed by Guihéneuf et al. (2014). Zinc analyses were performed with an ICP-OES (ThermoFisher, Scientific Walthan, MA). The device was operated at RF power: 1,300 W, argon plasma flow rate: 8 L.min^−1^, auxiliary argon flow rate: 0.3 L min^−1^, nebulizer argon flow rate: 0.80 L min^−1^ and sample flow: 1.0 L min^−1^. Zinc was read on axial mode at 213.857 nm.

## Results

### Enrichments

#### Sulfidogenic Activity

**Figure 1:**
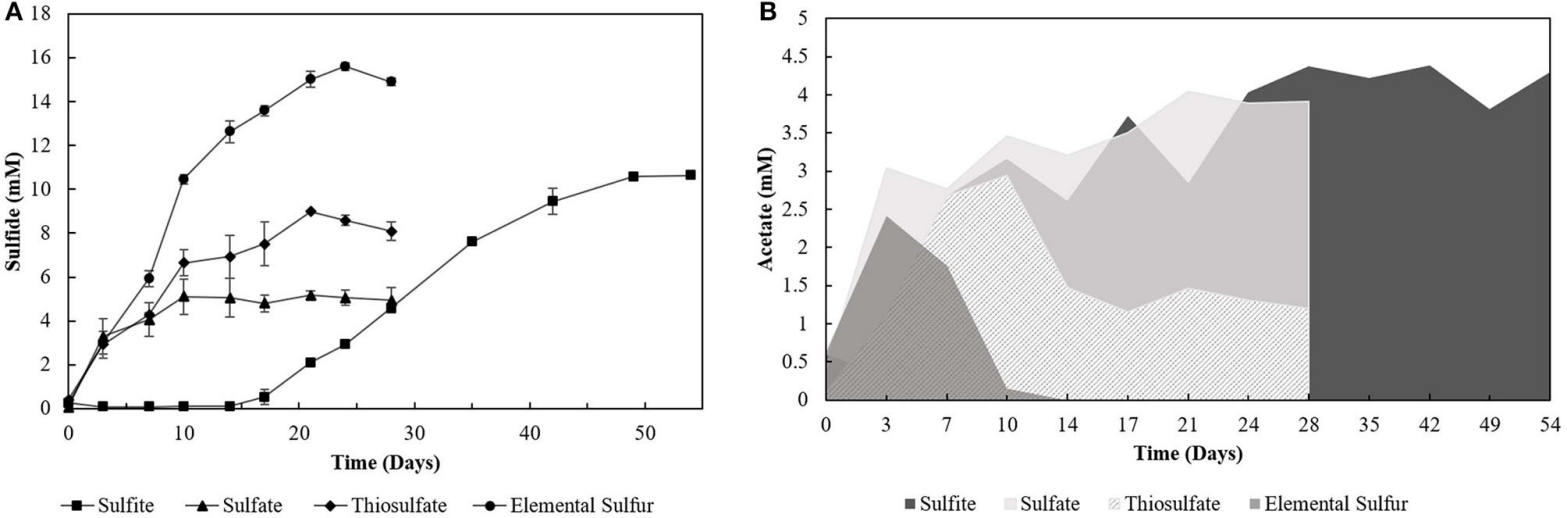
**(A)** Sulfide production and **(B)** acetate profile of the 4th transfer enrichments oxidizing oleate with sulfur compounds as electron acceptors. Measurements of biological triplicates were averaged, and the standard deviation is shown.

The degradation of oleate by the enrichments (4th transfer) was accompanied by sulfide production with all the electron acceptors used (Figure 1A). Acetate (Figure 1B) and CO_2_ were the other products detected in the incubations. Traces of hydrogen (0-0.01 mM) were detected in the headspace of all the 4th transfer cultures. Methane was not detected in the headspace of any enrichment. The pH values in S^0^- and S_2_${\text{O}}_{3}^{2-}$-reducing enrichments ranged from 5.5 to 7.0, while there was stability around pH 6–6.4 in ${\text{SO}}_{4}^{2-}$-reducing enrichments, and a variation from pH 6–8 in ${\text{SO}}_{3}^{2-}$-reducing cultures.

Incubations with S^0^ and ${\text{SO}}_{3}^{2-}$ yielded the highest production of sulfide, 15.60 (± 0.17) mM and 10.62 (± 0.12) mM, respectively. However, ${\text{SO}}_{3}^{2-}$-reducing enrichments presented a lag phase of 14 days, and acetate accumulated at 4.29 (± 0.28) mM, while it got completely depleted within 14 days of incubation in the S^0^ -reducing enrichment. Enrichments incubated with ${\text{SO}}_{4}^{2-}$ as electron acceptor produced maximally 5.16 (± 0.19) mM sulfide, with an accumulation of 4.05 (± 0.14) mM of acetate. The concentration of sulfate decreased by 7.44 (± 1.60) mM. Enrichments growing on S_2_${\text{O}}_{3}^{2-}$ produced up to 8.99 (± 0.02) mM of sulfide, and acetate accumulated at 1.20 (± 0.09) mM with a S_2_${\text{O}}_{3}^{2-}$ consumption of 5.57 (± 1.16) mM. In control groups, sulfide production was detected in the first transfer (4.07 ± 0.18 mM) due to the endogenous electron donors present in the inoculum sludge. In further transfers, sulfide production in control groups was no longer detected.

#### Microbial Communities

**Figure 2:**
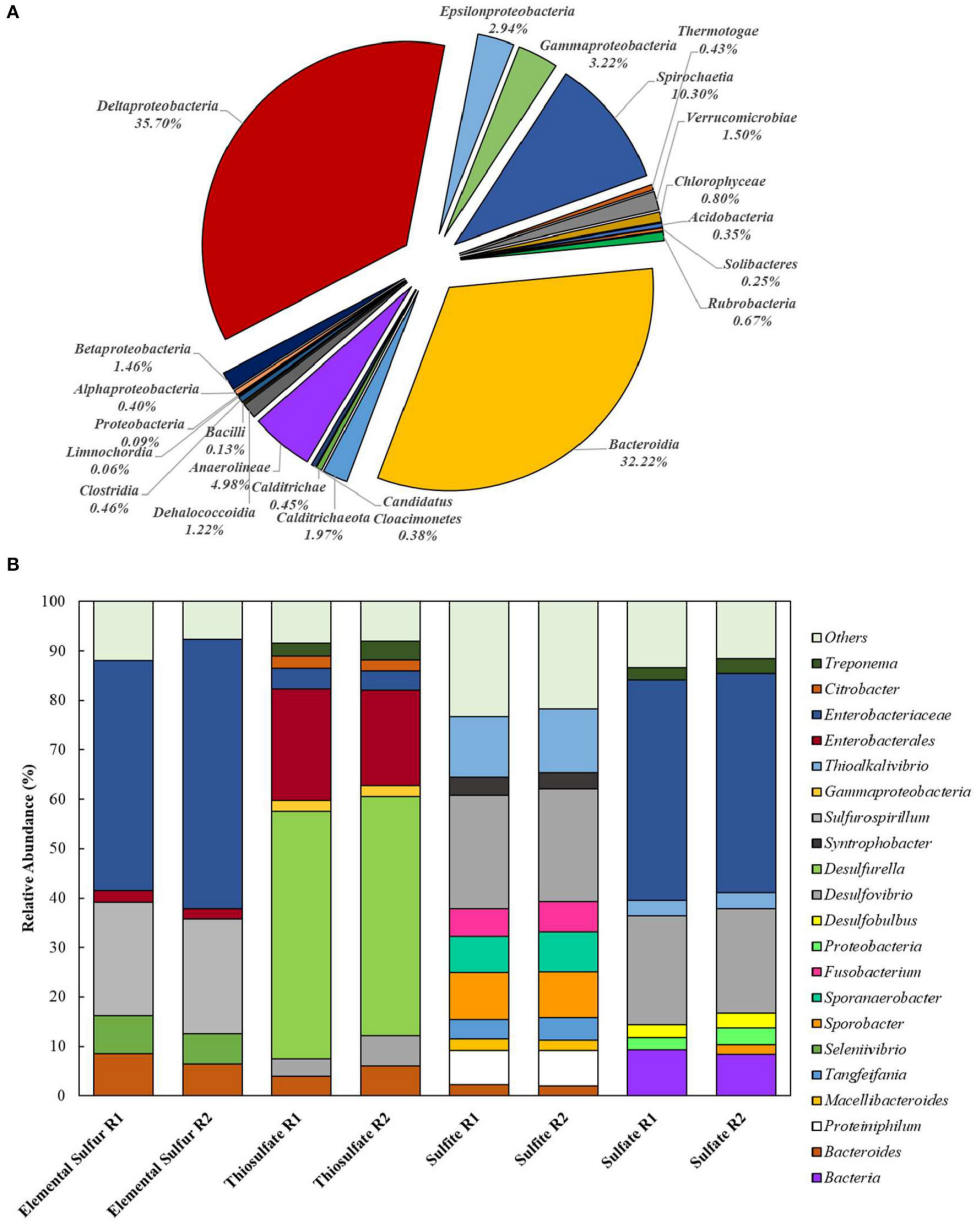
Bacterial diversity in the **(A)** inoculum (at the class level) and in the **(B)** 4th transfer enrichments on early stationary phase (at the genus level). Groups that could not be identified at the mentioned level were classified at the next highest possible resolution level. OTUs with less than 2% sequence reads were combined in the group “Others.” R1 – replicate 1; R2 – replicate 2.

Extracted DNA from the enrichments showed amplification only with bacterial primers, indicating that archaeal communities did not develop in the cultures. Enriched cultures were analyzed in duplicate per electron acceptor used. Summarized statistics of the 16S rDNA gene amplicon analysis of the inoculum source and enriched cultures are given in Table 1. After quality processing, sequences from the inoculum assigned to OTUs represented 21 known classes, with 79 correlated genera. The three most representative classes were *Deltaproteobacteria* (35.7% of the sequences), *Bacteroidia* (32.2% of the sequences), and *Spirochaetia* (10.3% of the sequences) (Figure 2A). About 2.8% of the total number of sequences could not be identified at the class level and were classified at the phylum level.

**Table 1:** Statistics of the extracted DNA analysis for the tested conditions.

**Sample**	**1**	**2**	**3**	**4**
Inoculum	84,356	92.8%	57.7%	423
S^0^ (R1)	59,731	98.8%	81.6%	422
S^0^ (R2)	1,185,202	96.7%	83.3%	427
S_2_${\text{O}}_{3}^{2-}$ (R1)	67,425	96.3%	77.1%	428
S_2_${\text{O}}_{3}^{2-}$ (R2)	84,966	96.4%	77.7%	428
${\text{SO}}_{3}^{2-}$ (R1)	78,925	95.7%	68.8%	422
${\text{SO}}_{3}^{2-}$ (R2)	78,972	94.6%	66.0%	422
${\text{SO}}_{4}^{2-}$ (R1)	72,254	97.5%	79.9%	427
${\text{SO}}_{4}^{2-}$ (R2)	78,711	98.4%	81.2%	427

*1) Input sequences, 2) Sequences after quality processing, 3) Sequences assigned to OTUs, 4) Length of the nucleotides. R1 – replicate 1, R2 – replicate 2*.

In enriched cultures, sequences that could not be identified at the genus level were classified at the next highest possible resolution level. In S^0^-reducing cultures, there was a predominance of *Enterobacteraceae* (50.6 ± 5.7%), *Sulfurospirillum* (23.1 ± 0.1%), *Bacteroides* (7.4 ± 1.5%), and *Seleniivibrio* (6.9 ± 1.1%). In S_2_${\text{O}}_{3}^{2-}$-reducing enrichments, the genus *Desulfurella* predominated in about 49.2 (± 1.2) % of the total number of sequences, followed by 20.9 (± 2.3)% of *Enterobacterales*. ${\text{SO}}_{3}^{2-}$-reducing cultures presented the highest diversity, with 10 OTUs representing more than 2% of the total number of sequences. From these, 22.9 (± 0.1) % were represented by the genus *Desulfvibrio*, followed by 12.6 (± 0.5) % of *Thioalkalivibrio*. In cultures that received sulfate as electron acceptor, *Enterobacteraceae* dominated the samples with 44.4 (± 0.2) % of the sequences, followed by *Desulfovibrio* with 21.5 (± 0.6) % of the sequences.

### Oleate Tolerance Tests

**Figure 3:**
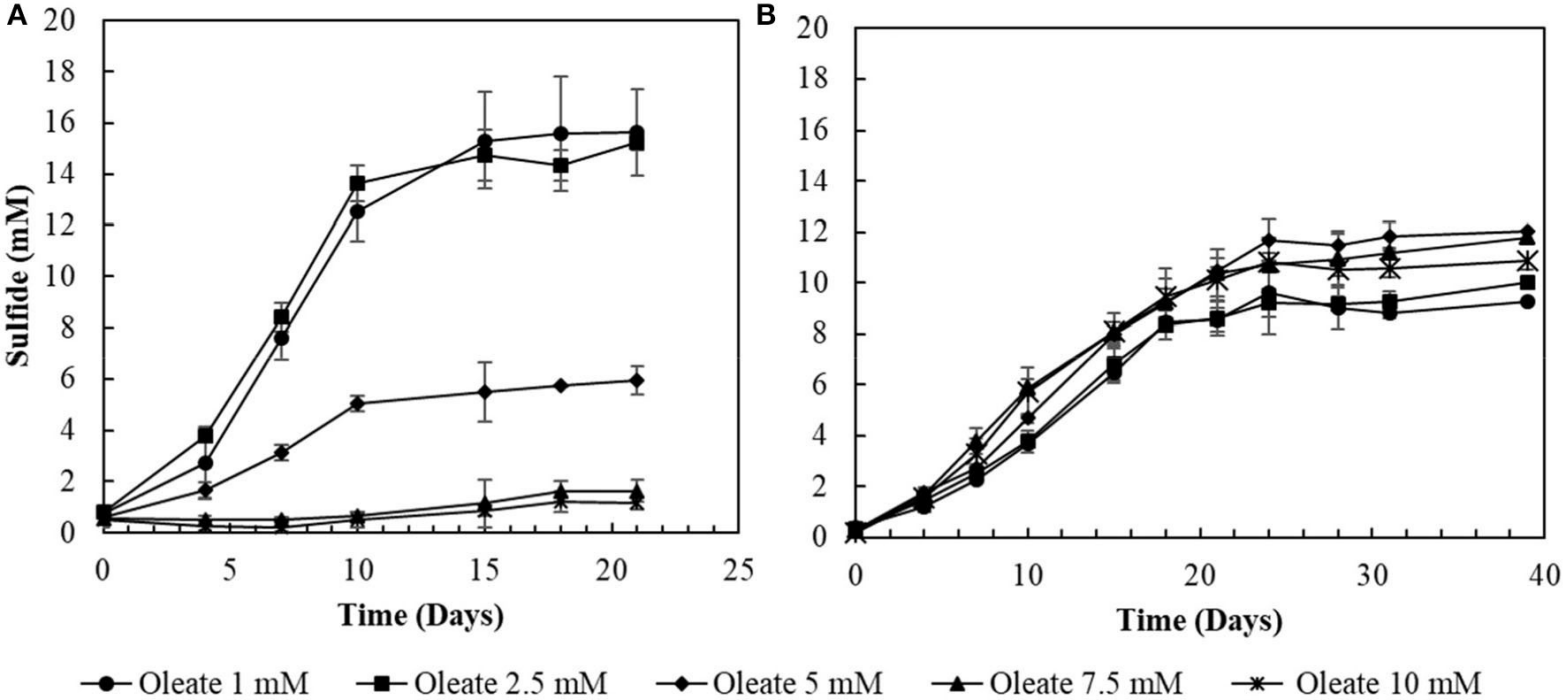
Toxicity profiles of oleate to **(A)** elemental sulfur- and **(B)** thiosulfate-reducing cultures. Measurements of biological triplicates were averaged, and the standard deviation is shown.

The two conditions with the best combined sulfidogenic performance, with the minimal acetate accumulation and with a more specialized microbial community (S^0^- and S_2_${\text{O}}_{3}^{2-}$-reducing enrichments) were selected for further analyses. Increasing concentrations of oleate in the medium affected the sulfide production by S^0^-reducing cultures (Figure 3A). Concentrations of oleate of 1 and 2 mM did not impact the sulfide production by the enrichments. However, when it was added to a final concentration of 5 mM, the sulfidogenic activity of the cultures decreased by more than 50%, and triplicates receiving 7.5 mM of oleate did not show significant sulfide production when compared to the initial concentrations.

In S_2_${\text{O}}_{3}^{2-}$-reducing cultures, the increase in oleate concentrations in the medium initially promoted an improvement in sulfide production, from 9.3 (± 0.2) mM in 1 mM oleate condition to 12.0 (± 0.6) mM in the 5 mM oleate condition. Although oleate concentrations exceeding 5 mM still promoted sulfide concentrations higher than at 1 mM, there was a decrease in comparison to the optimum obtained with 5 mM of sodium oleate (Figure 3B). In the 7.5 mM oleate culture, the production of sulfide reached 11.8 (± 0.2) mM, and, when oleate was supplied to a final concentration of 10 mM, the sulfide production decreased to 10.9 (± 0.3) mM (Figure 3B).

### LCFA Degradation Profile

**Figure 4:**
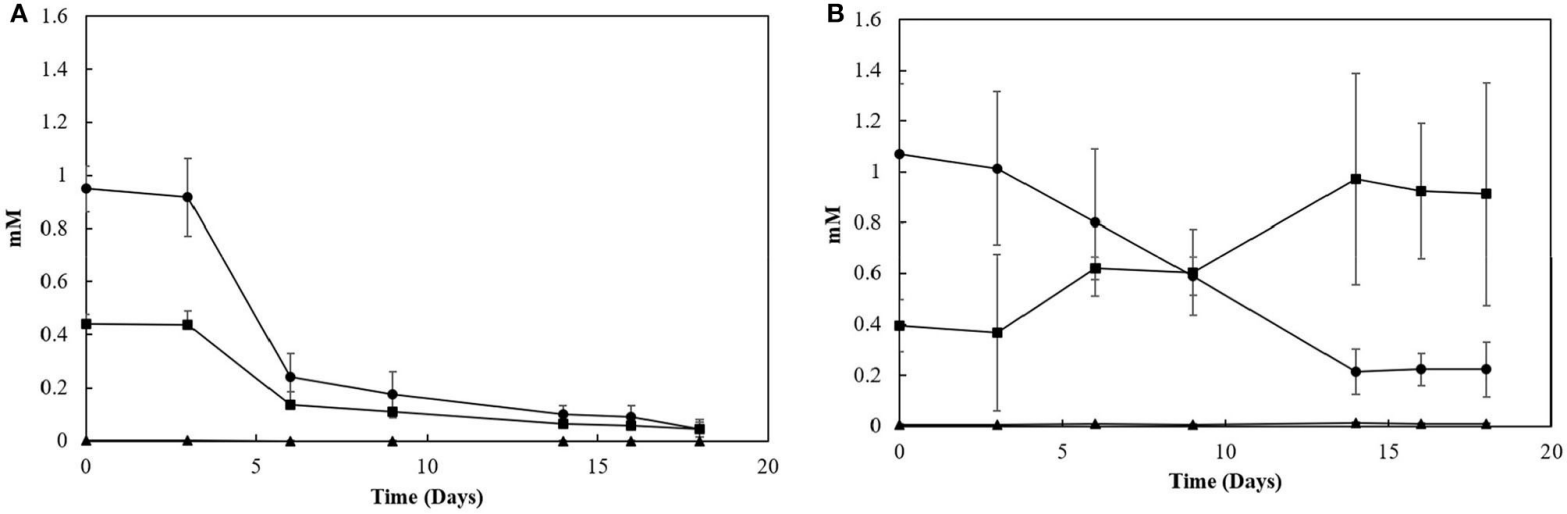
LCFA profiles in sacrificial experiments with cultures growing with **(A)** elemental sulfur or **(B)** thiosulfate as electron acceptors. Measurements of biological triplicates were averaged, and the standard deviation is shown. • oleate, ■ palmitate, ▴ myristate.

**Figure 5:**
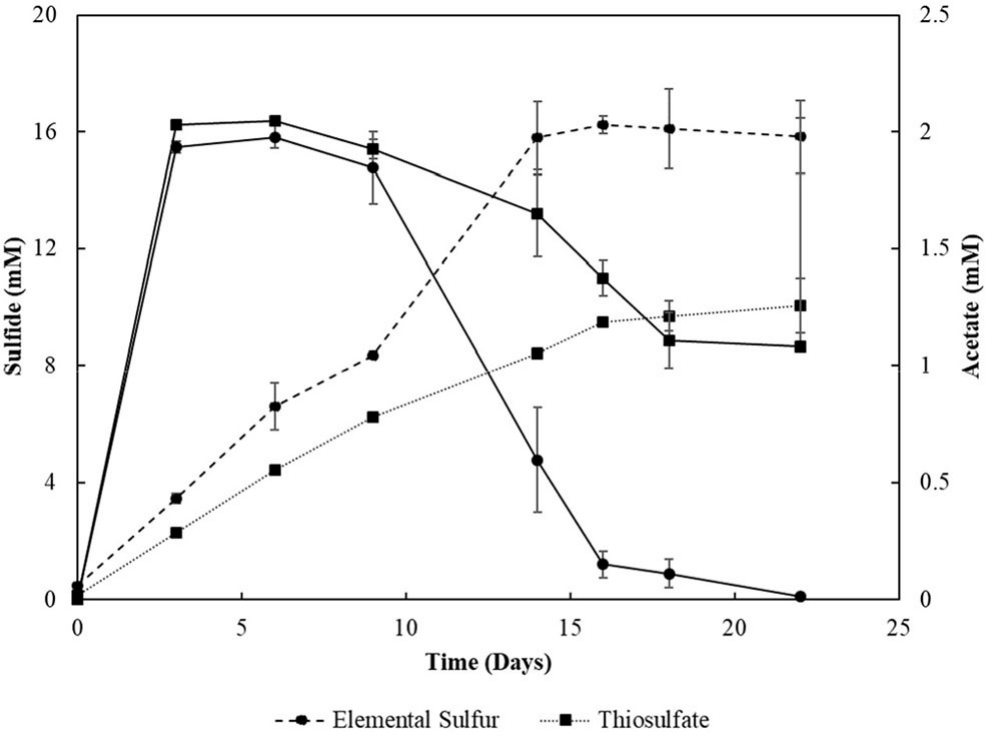
Sulfide production and acetate profile of the enrichments oxidizing oleate with elemental sulfur or thiosulfate as electron acceptors in sacrificial experiments. Dashed lines correlate to sulfide, and continuous lines correlate to acetate concentrations. Measurements of biological triplicates were averaged, and the standard deviation is shown.

Sacrificial experiments were performed to investigate the degradation profile of oleate by the enrichments (4th transfer) in the presence of S^0^ and S_2_${\text{O}}_{3}^{2-}$. The S^0^-reducing enrichments degraded 0.9 (± 0.1) mM of oleate in 22 days, with no accumulation of palmitate or any other LCFA analyzed (Figure 4A). The acetate concentration peaked at 2.0 (± 0.0) mM in 6 days of experiment but got completely depleted within 22 days. The sulfide production reached 15.8 (± 1.3) mM (Figure 5). In S_2_${\text{O}}_{3}^{2-}$-reducing enrichments, 0.8 (± 0.2) mM of oleate was consumed in 14 days, while 0.5 (± 0.3) mM of palmitate was produced. The concentration of myristate did not significantly change along the sacrificial experiment (Figure 4B). Laureate was not detected at any point of the growth curve. In these S_2_${\text{O}}_{3}^{2-}$ -fed enrichments, 10.0 (± 0.9) mM of sulfide was produced, while a peak of acetate was observed on day 6 (2.0 ± 0.0 mM) and decreased to 1.1 (± 0.1) mM at the end of the incubation (Figure 5).

### Performance of Sequencing Batch Reactors

**Figure 6:**
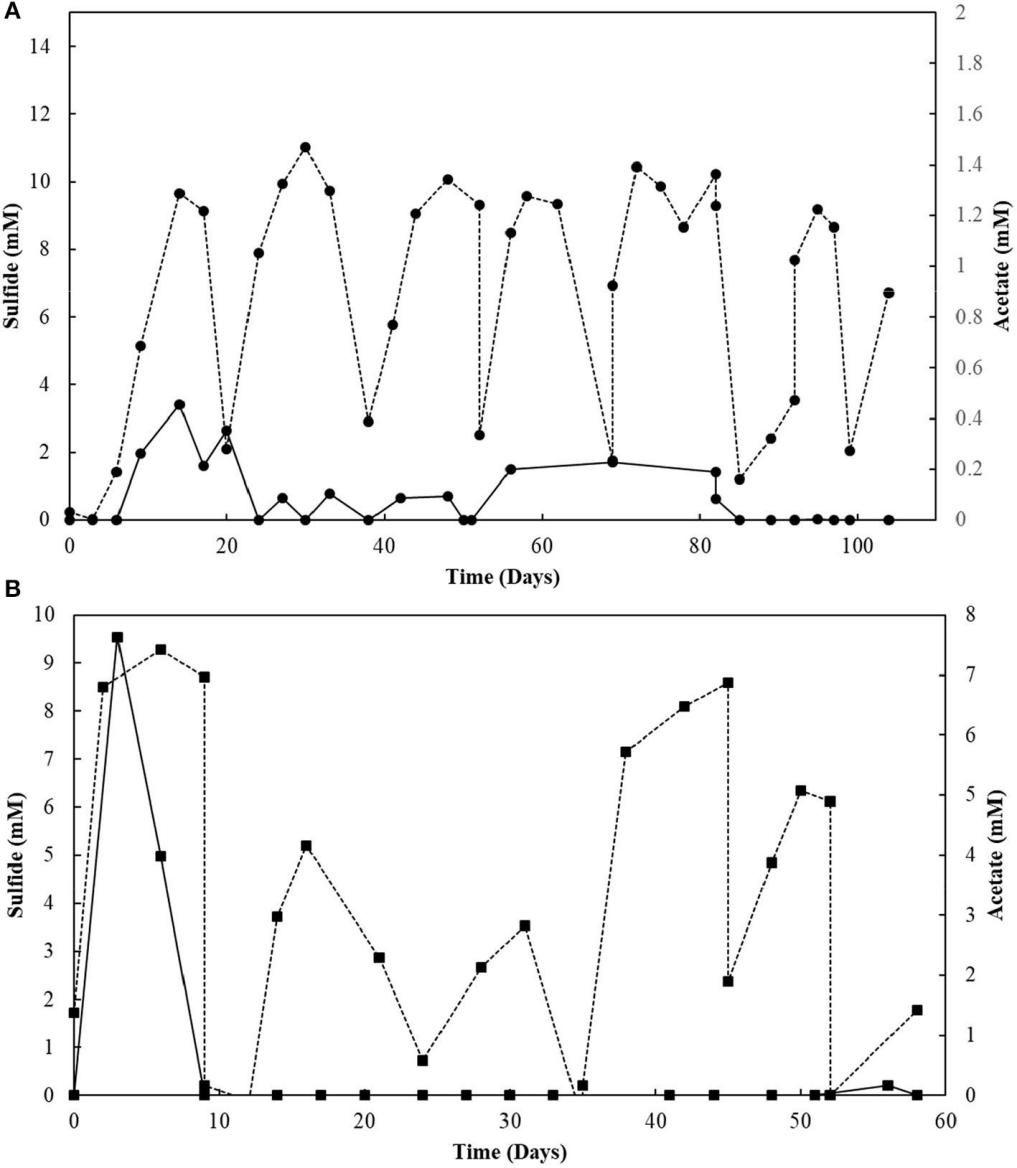
**(A)** Elemental sulfur and **(B)** Thiosulfate reactors performance operating in sequencing batch with sulfide stripping. Dashed lines correlate to sulfide, and continuous lines to acetate concentrations.

The elemental sulfur reactor was operated for 104 days (Figure 6A). Every ± 17 days, when sulfide reached the plateau phase (10.0 ± 0.6 mM), it was stripped from the system. Its production was subsequently restored, coupled to the degradation of oleate, replenished every about 39 days to a concentration of 2 mM. Acetate was transiently detected in the reactor mixed liquor and its concentration reached a maximum of 0.4 mM. The sulfide accumulated till day 39 was stripped and introduced to an acidic ZnCl_2_ solution, where about 73.4 mM of zinc precipitated as ZnS. The sulfide accumulated during the second cycle before replenishment of the medium (day 92) resulted in a total of 51.4 mM of zinc precipitated as ZnS.

**Figure 7:**
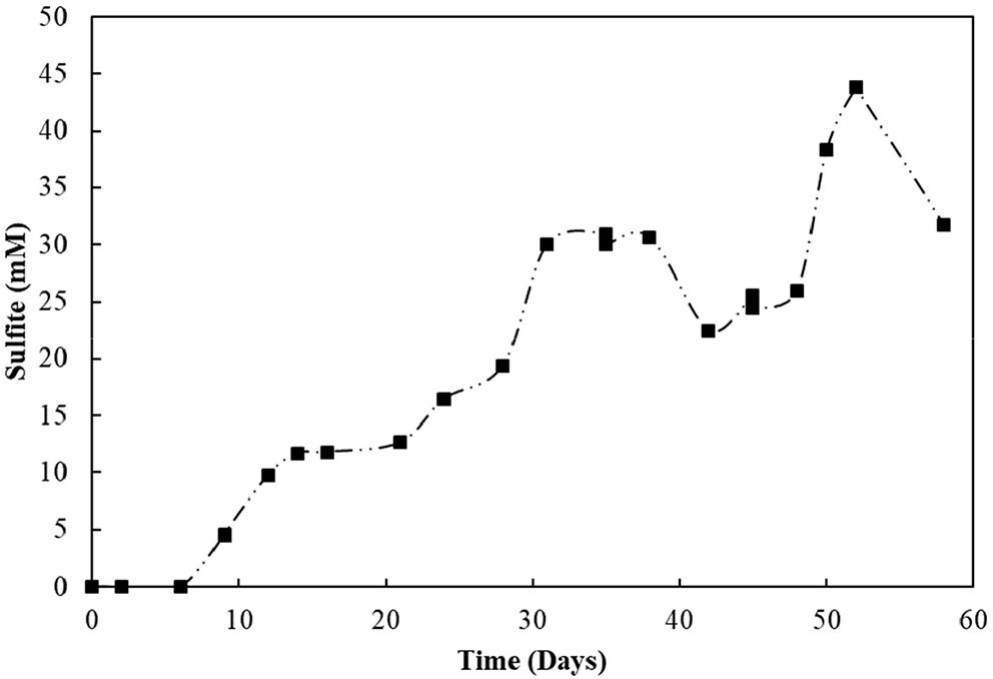
Sulfite accumulation in the reactor with thiosulfate-reducing culture.

The S_2_${\text{O}}_{3}^{2-}$-reducing bioreactor was operated for 58 days (Figure 6B). Sulfide production reached 9.3 mM in the first 6 days of reactor operation. However, after the first gas stripping, the sulfide production suffered a significant decrease, reaching maximally 5.2 mM in the next 30 days of operation, after which it increased up to 8.6 mM on day 45, and decreased again. The acetate concentration peaked at 7.6 mM within 3 days of operation, with full depletion in 9 days. After 51 days of operation, the acetate concentration slightly raised in the reactor. From the initial 50 mM of thiosulfate fed to the sequencing batch reactor, 17.2 mM was left after 58 days of operation, and the sulfite concentration of the reactor mixed liquor increased up to 43.8 mM within 52 days (Figure 7). The accumulated sulfide stripped precipitated a total of 23.4 mM of Zn as ZnS in the first cycle of sulfide stripping, while the cumulative sulfide in the second cycle precipitated as 49.4 mM ZnS.

**Figure 8:**
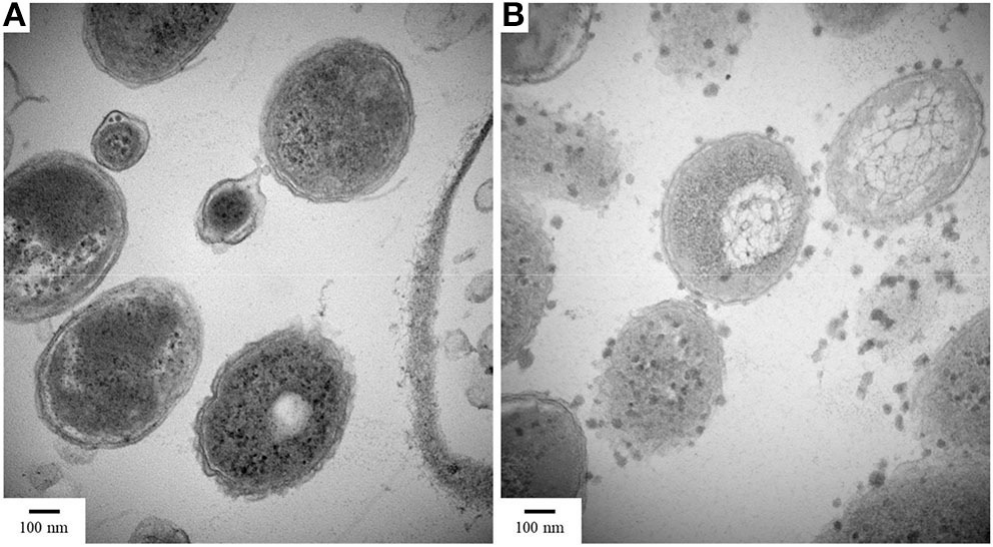
TEM images of cultures growing with oleate and **(A)** elemental sulfur or **(B)** thiosulfate. Samples were taken on day 50 of the reactors' operation. Magnification 80,000x.

**Figure 9:**
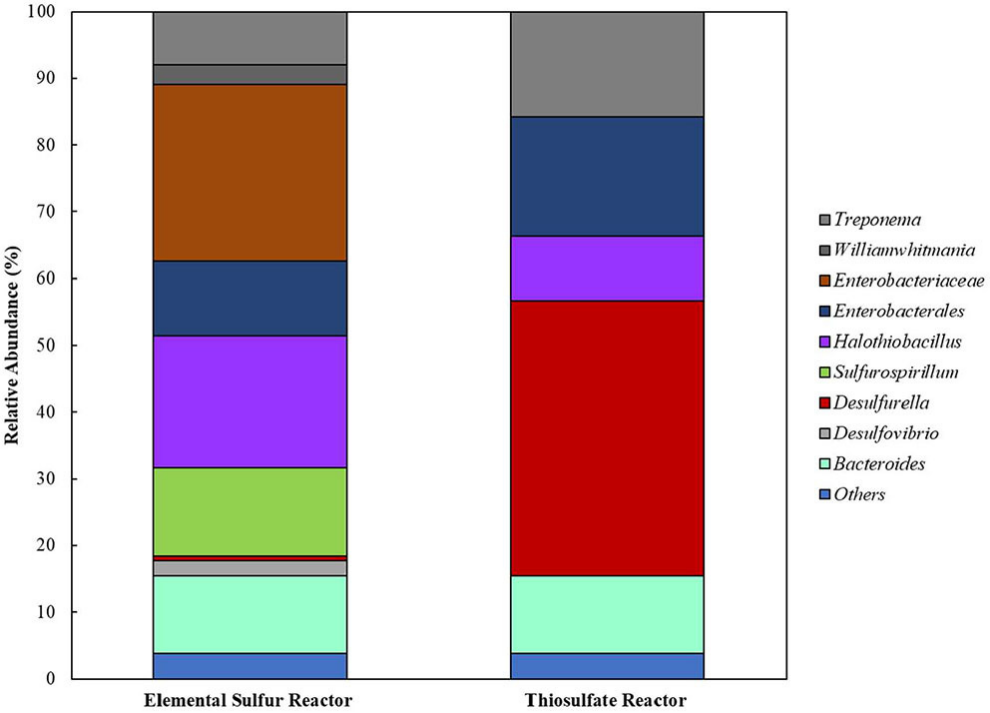
Bacterial diversity in the reactors oxidizing oleate with elemental sulfur or thiosulfate as electron acceptors. OTUs with <2% sequence reads were combined in the group “Others”.

When cross-sectional TEM analysis was performed on the reactors' biomass, a distinct pattern of interaction between S^0^/ S_2_${\text{O}}_{3}^{2-}$ -fed bacterial cells and LCFA can be hypothesized. S^0^-reducing cells likely internalized the LCFA as particles, visible as the intracellular dark dots (Figure 8A), while S_2_${\text{O}}_{3}^{2-}$-reducing microorganisms seemed to perform the degradation on the surface of their membranes (Figure 8B). Analysis of the microbial diversity shows, as for the enrichments, a greater diversity of OTUs in the reactor operating with elemental sulfur as electron acceptor (Figure 9). In the biomass present in this reactor, sequences belonging to the family *Enterobacteraceae* represented 26.5% of the diversity, followed by the genera *Halothiobacillus, Sulfurospirillum*, and *Bacteroides*, with 19.7, 13.3, and 11.5%, respectively. In the reactor fed with thiosulfate as electron acceptor, the bacterial diversity was greatly dominated by the genus *Desulfurella*, with 41.3% of the sequences. The order *Enterobacterales* and the genus *Bacteroides* comprised the second (17.9%) and third (11.6%) OTUs better represented in the thiosulfate-fed reactor.

### Sulfite Toxicity

**Figure 10:**
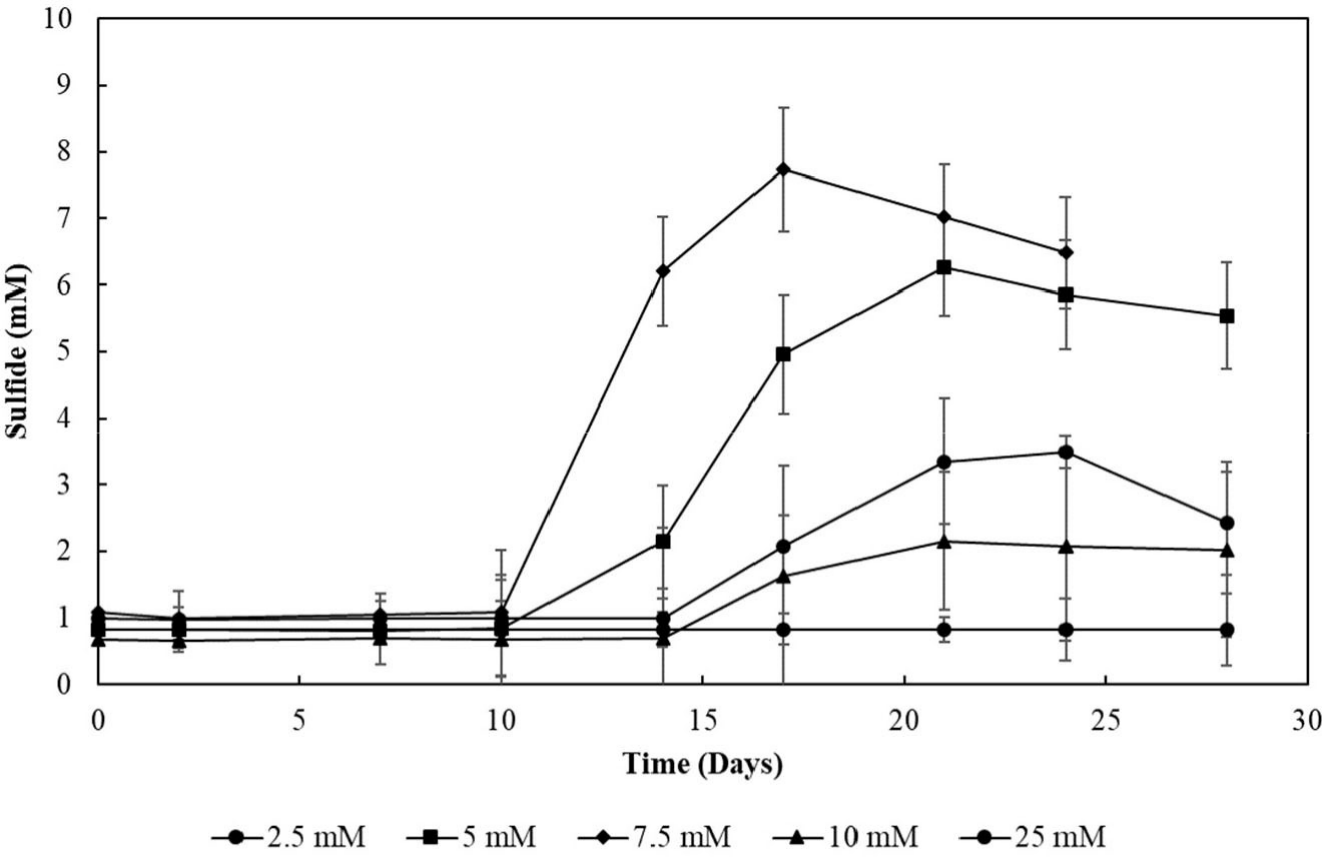
Evolution of sulfide production in cultures incubated with increasing sulfite concentrations. Measurements of biological triplicates were averaged, and the standard deviation is shown.

The decrease in sulfide production in the thiosulfate-reducing reactor motivated the investigation of sulfite toxicity to the biomass. When S_2_${\text{O}}_{3}^{2-}$–SBR biomass was incubated with increasing sulfite concentrations, a great impact on the sulfide production was confirmed (Figure 10). When sulfite was supplied to the S_2_${\text{O}}_{3}^{2-}$–SBR biomass in initial concentrations up to 7.5 mM, the production of sulfide was equivalent to the concentration of the electron acceptors. When 10 mM of sulfite was used, sulfide generation by the S_2_${\text{O}}_{3}^{2-}$–SBR biomass drastically decreased, reaching maximally 1.5 (± 1.0) mM. At an initial concentration of 25 mM, sulfide was no longer produced by the S_2_${\text{O}}_{3}^{2-}$–SBR biomass.

## Discussion

### Electron Acceptor Screening

This study showed that incubation of anaerobic sludge treating dairy wastewater with oleate and sulfur compounds diverted the fluxes of electrons from methanogenesis toward sulfide production, leading to different enrichments of sulfur compounds-metabolizing cultures. To the best of our knowledge, this is the first report in which different sulfur compounds were screened for oleate-degradation coupled to sulfidogenic capability.

Although not reaching the maximum amount of sulfide expected per mM of electron donor added, the sulfidogenic capacity of the enrichments and reactors' biomass followed the proposed stoichiometric reactions for each compound (Equations 1–4). The lag phase observed in the ${\text{SO}}_{3}^{2-}$-reducing enrichments (Figure 1A) reflects the possible toxic effect of sulfite to the cells, in accordance with previously reported observations (Irwin et al., 2017). Together with the ${\text{SO}}_{4}^{2-}$-reducing enrichments, they showed the highest acetate accumulation (Figure 1B), indicating an incomplete oxidation metabolism by the enriched microorganisms.

(1) $$\begin{array}{r} C_{18}H_{33}O_{2}^{-}+51S^{0}+34H_{2}O\;\to 51S^{2-}+18CO_{2}+101H^{+} \end{array}$$

(2) $$\begin{array}{r} C_{18}H_{33}O_{2}^{-}+17SO_{3}^{2-}+H^{+}\to 17S^{2-}+17H_{2}O+18CO_{2} \end{array}$$

(3) $$\begin{array}{r} C_{18}H_{33}O_{2}^{-}+12.75SO_{4}^{2-}+H^{+}\to 12.75S^{2-}+17H_{2}O+18CO_{2} \end{array}$$

(4) $$\begin{array}{r} C_{18}H_{33}O_{2}^{-}+12.75S_{2}O_{3}^{2-}\;\to 25.5S^{2-}+4.25H_{2}O+18CO_{2}+24.5H^{+} \end{array}$$

The limitation in sulfide production observed in all the enrichments might have provoked a slighter decrease in pH than expected by the above-shown equations, allowing the viability of the cells. The decrease in pH to a minimum of 5.0 is also an important feature of the enrichments, as this does not allow dissociation of acetate to acetic acid >50% (pKa = 4.75), which would acidify the medium and inhibit the microorganisms.

The addition of LCFA and sulfur compounds to anaerobic sludge was also described by Sharma and Biswas (2013), in which linoleic acid was used to inhibit methanogenic activity and enhance sulfate reduction in the sludge. Although the presence of sulfate in methanogenic environments thermodynamically favors the sulfidogenic reaction, in that study the authors observed that the diversion of electron fluxes (in more than 30%) was a function of the LCFA concentration. Salvador et al. (2019) investigated the degradation of oleate by an anaerobic culture acclimated to methane production. In the presence of a sulfonate with methanogenic-inhibiting activity (isethionate), sulfate-reducing bacteria capable of sulfonate metabolism got enriched and started a synergistic activity with oleate-degrading bacteria with conversion of the LCFA to acetate. In both studies, only sulfate was tested as electron acceptor for sulfide production.

The bacterial diversity obtained in the enrichments (Figure 2) reveals the great potential for sulfur metabolism in the anaerobic sludge treating dairy products. Besides, the metabolic flexibility of microorganisms able to reduce sulfur compounds is well-known (Plugge et al., 2011; Rabus et al., 2013; Florentino et al., 2016b). S_2_${\text{O}}_{3}^{2-}$-reducing enrichments revealed the greatest bacterial specialization, with the predominance of members of the *Desulfurella* genus. This genus is known for its ability of reducing elemental sulfur as its major metabolism, coupled to the oxidation of simple and complex organic matter (Bonch-Osmolovskaya et al., 1990; Miroshnichenko et al., 1998; Florentino et al., 2016a). Although it has been reported that *Desulfurella* species can grow on LCFA, there are no reports of growth curves or physiological studies of this genus directly degrading LCFA for sulfide production. The second predominance was of the order *Enterobacterales*, which has not been reported as a group of sulfur metabolizers as major metabolism. This opens possibilities for isolation of novel microorganisms from this order with great ability for sulfide production coupled to degradation of FOG. A second possibility is, however, that members of the *Enterobacterales* played a role as LCFA-degrading groups (Odeyemi et al., 2013; Liu et al., 2018), not coupled to the reduction of thiosulfate, but generating acetate and hydrogen to be used as electron donors for *Desulfurella* members, as acetate is the best electron donor for these species (Schmitz et al., 1990; Florentino et al., 2016a).

The *Enterobacteraceae* family was predominant in S^0^-reducing enrichments, followed by the *Desulfovibrio* genus (Figure 2). The presence of *Desulfovibrio* species with elemental sulfur as electron acceptor opens possibilities for further studies, as there are no reports of extended sulfidogenesis from the reduction of sulfur coupled to the degradation of complex organic matter by this group. Escobar et al. (2007) showed the feasibility of elemental sulfur reduction by *Desulfovibrio desulfuricans* with hydrogen as electron donor and obtained maximal sulfide production rates of 2.1 g H_2_S L^−1^ d^−1^. However, Biebl and Pfennig (1977) tested *D. desulfuricans* and *D. vulgaris* and no growth on elemental sulfur was observed, but slow and definite growth was observed for *D. gigas*. As the *Enterobacteraceae* family was highly predominant, it is also reasonable to hypothesize that the LCFA degradation in those enrichments was not directly coupled to elemental sulfur reduction, but the acetate produced from the β-oxidation chain reaction was likely the actual electron donor for the *Desulfovibrio* species for the sulfur-reducing process.

Despite the great diversity observed in ${\text{SO}}_{3}^{2-}$-reducing enrichments (Figure 2), members of the *Thioalkalivibrio* and *Desulfovibrio* genera showed relative predominance. The pronounced enrichment of *Thioalkalivibrio* members in the tested conditions is unexpected, as this haloalkaliphilic group, belonging to the order of Purple Sulfur Bacteria, is commonly reported as sulfide and/or elemental sulfur oxidizers (Sorokin and Kuenen, 2005; Ahn et al., 2017). Therefore, the enrichment of *Thioalkalivibrio* requires further investigation focused on the isolation and characterization of the potential novel species able to thrive under sulfidogenic conditions. As above-mentioned, it could be that the presence of *Desulfovibrio* was correlated to the release of acetate and hydrogen by the β-oxidation chain reaction performed by LCFA-degrading groups, such as *Syntrophobacter*, also identified in this enrichment (Figure 2). However, the accumulation of acetate in ${\text{SO}}_{3}^{2-}$-reducing enrichment suggest that *Desulfovibrio* members might perform incomplete oxidation in this incubation, and therefore such members would not be able to use acetate as electron donor, only hydrogen. Further investigation is required to clarify the role played by the enriched groups in LCFA degradation.

Like in the S_2_${\text{O}}_{3}^{2-}$-reducing enrichments, members of the family *Enterobacteraceae* represented the great majority of the identified groups in ${\text{SO}}_{4}^{2-}$-reducing cultures, but, in this condition, it was followed by members of the *Desulfovibrio* genus. Sousa et al. (2009) also observed an enrichment of members of the genus *Desulfovibrio* in oleate-degrading cultures incubated with sulfate as electron acceptor. The latter authors associated the presence of the *Desulfovibrio* species to the release of acetate and hydrogen by the β-oxidation chain reaction performed by syntrophic bacterial groups. However, as mentioned for ${\text{SO}}_{3}^{2-}$-reducing enrichments, acetate accumulation could be an indication of the incomplete oxidation metabolic route in the enriched *Desulfovibrio* species, with hydrogen as the only electron donor in this case.

### LCFA Degradation in S^0^ vs. S_2_${\text{O}}_{3}^{2-}$ -Fed SBRs

The sulfidogenic activity of the S^0^-reducing reactor biomass was affected by increasing oleate concentrations, with negative effects starting from 5 mM (Figure 3). In contrast, biomass from the thiosulfate-reducing reactors did not suffer a great impact from concentrations of oleate up to 10 mM. There are no reports about the effects of oleate on sulfidogenic pure cultures or bioreactor sludge. On methanogenic communities, however, the inhibitory effects of oleate already occur at concentrations ranging from 0.3 to 2.3 mM (Hwu and Lettinga, 1997; Sousa et al., 2013; Silva et al., 2016). The higher tolerance of the sulfidogenic biomass to oleate reveals a noticeably higher resilience of sulfidogenic communities than methanogenic communities. Such resilience makes sulfidogenic dominated biomass an approachable surrogate for methanogenic groups for the degradation of LCFA, if the sulfide produced can be properly managed, as [e.g., by using for metal precipitation (see below)].

Furthermore, although the reactors' biomass was able to oxidize oleate, its degradation profile in the reactors did not follow the same pattern (Figure 4). In the S^0^-reducing enrichment, the decrease in oleate concentration in the medium was not followed by a peak of palmitate, myristate or any other LCFA that can be detected by the analytical method applied. Although the electron donor was not fully consumed (residual oleate concentration after 18 days: 0.9 mM), the sulfide production (16.1 mM) did not correlate to the observed decrease in oleate concentration. However, the presence of acetate reinforces the degradation of oleate (and the residual palmitate) initially present in the medium. Therefore, it is likely that the insolubility of elemental sulfur might have interfered with the LCFA extraction method, hampering the detection of the intermediate products and consequently the oleate degradation profile.

In S_2_${\text{O}}_{3}^{2-}$-reducing enrichments, however, the concentration of palmitate gradually increased, while no change in myristate or laureate concentration was observed (Figure 4B). Considering the amount of oleate and palmitate consumed, assuming that all the other long and short chain fatty acids were consumed and accounting the acetate left in the medium, about 3 mM of acetate was consumed by the enrichment, which would contribute to the production of ~6 mM of sulfide, less than the observed concentration (16.1 mM, Figure 5). The exceeding sulfide produced might have been produced by the hydrogen released by the β-oxidation process. However, no accurate calculation can be made.

Sulfidogenic enrichments performed on oleate did not show sulfide production any close to the stoichiometric reaction (Table 2). Two possible explanations for this observation are the maximal tolerance of the cultures to sulfide and/or the increasing concentration of palmitate in the medium as an intermediate product of oleate degradation. Although the literature reports negative effects of hydrogen sulfide on various microorganisms, including environmental bacteria (Beauchamp et al., 1984), there are only a few reports on the effects of this compound on its own producers. In general, the proposed sulfide concentration to inhibition of growth of sulfate-reducing bacteria is about 16 mM (Reis et al., 1992). Specific values for elemental sulfur- or thiosulfate-reducing cultures are not reported. As a second explanation, the accumulation of non-degraded LCFA in the cultures can also be a key factor for growth and activity limitation in methanogenic cultures (Pereira et al., 2005; Sharma et al., 2015). The adsorption of LCFA on the cell surface (Figure 8) is a possible explanation for physical inhibition of the bacterial community (Pereira et al., 2005). Besides, the differences in the composition of the cell wall might exert an influence on the sensitivity of microorganisms to the LCFA mixture present in the medium, which might play a selective role in the community (Silva et al., 2016).

**Table 2:** Expected and observed sulfide production from the oleate degradation coupled to elemental sulfur and thiosulfate reduction in the enriched bacterial communities during the screening experiment (4th transfer only).

**Stoichiometric reactions**	**Oleate degradation (mM)**	**Sulfide production (mM)**	
		**Expected**	**Observed**
$C_{18}H_{33}O_{2}^{-}+51S^{0}+34H_{2}O\to 51S^{2-}+18CO_{2}+101H^{+}$	0.9 ± 0.0	45.9	16.1 ± 1.2
$C_{18}H_{33}O_{2}^{-}+12.75S_{2}O_{3}^{2-}\to 25.5S^{2-}+4.25H_{2}O+18CO_{2}+24.5H^{+}$	0.8 ± 0.0	21.7	10.0 ± 0.9

### Sulfide vs. Sulfite Toxicity

The removal of hydrogen sulfide from the bioreactors by gas stripping shifted the reaction toward more LCFA degradation and, consequently, more sulfide production (Figure 6A). Moreover, the total sulfide produced from 2 mM of oleate was close to that stoichiometrically expected for the conversion of oleate to palmitate in the β-oxidation chain reaction, which indicates the degradation of palmitate. Thus, our hypothesis of growth and activity inhibition due to elevated sulfide concentrations was reinforced.

This observation was less pronounced in the thiosulfate reactor (Figure 6B). Elevated initial concentrations of thiosulfate as electron acceptors for the biomass in the reactor promoted a high production of sulfite, as an intermediate product of thiosulfate reduction (Equation 5). Typically, the reductive process of thiosulfate is reported to happen in two steps. First, thiosulfate is converted to sulfide and sulfite. The sulfide formed leaves the cell, while the sulfite enters the cytoplasm and gets reduced to sulfide. Overall, one sulfide is generated during the first step, directly from thiosulfate molecule, and the other is generated in the second step, during sulfite reduction (Surkov et al., 2000; Stoffels et al., 2012; Florentino et al., 2019).

(5) $$\begin{array}{r} {\text{S}}_{2}{\text{O}}_{3}^{2-}+2{\text{H}}^{+}+2{\text{e}}^{-}\to \text{H}{\text{S}}^{-}+\text{HS}{\text{O}}_{3}^{-} \end{array}$$

Sulfite was toxic to the enriched cultures at initial concentrations of 10 mM (Figure 10), which is in accordance with the reactor's performance: the sulfide production in this case decreased by about 80% (Figure 6B). The increased concentration of sulfite likely imposed inhibition to the S_2_${\text{O}}_{3}^{2-}$-reducing cultures and a decreased sulfide production, motivating the sulfite toxicity experiments (discussed in previous section). It is worth noting that, in sulfidogenic reactors fed thiosulfate as electron acceptor, the thiosulfate influent concentration should thus be carefully monitored, as the production of sulfite as an intermediate depends on it and might cause damage to growth and sulfidogenic performance of the reactor.

Sulfite toxicity to cells has been widely reported (Daniel, 1986; Gardner et al., 2015; Kappler and Enemark, 2015; Kappler and Schwarz, 2016). Although this highly reactive compound is on one hand referred to damage proteins, DNA and lipids through the formation of adducts, and on the other hand sulfite-oxidizing enzymes (SOEs) are found in nearly all forms of life (Kappler and Enemark, 2015; Kappler and Schwarz, 2016), very little is known about its mechanism of toxicity toward anaerobic microorganisms.

### Metal Recovery

The aquatic environmental pollution originating from heavy metals in solution is of utmost relevance due to its impact on public health, environment, and economy. Sulfidogenic reactors have become suitable alternatives for the treatment of metal-rich wastewaters (Escobar et al., 2007; Gallegos-Garcia et al., 2009), with high levels of metals precipitated as highly insoluble metal sulfides. As a proof of concept, the stripping of sulfide for heavy metal precipitation shown in this study (Figure 6) increased the possibility of degrading the highly energetic bonds in LCFA compounds, mitigating the complexity of FOGs in lipid-rich wastewater while removing and recycling metals from metallurgical emissions.

Although the precipitation of metals with sulfide is widely implemented in several reactors configurations, the majority of the studies is performed with sulfate as electron acceptor and with simple organic compounds or hydrogen as electron donors (Jong and Parry, 2003; Kaksonen et al., 2004; Sierra-Alvarez et al., 2007; Gallegos-Garcia et al., 2009). The use of LCFA by mixed microbial communities with the flux of electrons drained toward sulfide production has, thus far, not received much attention. Sharma and Biswas (2013) applied sulfate as electron acceptor in a methanogenic culture fed with glucose in the presence of linoleic acid and observed a shift in the reaction toward sulfide production. However, the authors did not perform any further investigation on the utilization of the produced sulfide, or on the microbial community shift.

The reactor operating with thiosulfate kept the bacterial diversity, with *Desulfurella* and *Enterobacterales* as the most dominant groups. In the elemental sulfur-reducing reactor, although *Enterobacteraceae* and *Sulfurospirillum* members remained with good abundance, *Halothiobacillus* and *Bacteroides* members got enriched as well. To our knowledge, the abundance and diversity of sulfidogenic microorganisms in anaerobic reactors is limited, it is thus worthy to investigate how to further improve the efficiency of metal precipitation processes by applying specific conditions to enhance the growth and metabolic activity of these specialized microorganisms.

The sulfite toxicity to S_2_${\text{O}}_{3}^{2-}$-reducing enrichments observed in this study imposes some constraints to the broad utilization of sulfidogenic LCFA-degrading biomass for biotechnological purposes, when thiosulfate is present in elevated concentrations. The robustness and great efficiency of S^0^-reducing cultures, however, reveals a promising alternative to the currently applied strategies for the degradation of LCFA, with no accumulation of intermediate products and feasible destination of the end-product (H_2_S). It is, therefore, a good prospect for enhanced metal recovery and generation of clean industrial effluents.

## Data Availability Statement

The datasets generated for this study can be found in online repositories. The names of the repository/repositories and accession number(s) can be found at: https://www.ncbi.nlm.nih.gov/, PRJNA622591.

## Author Contributions

AF: study conception, experimental design, and drafting of manuscript. AF, RC, and YH: acquisition of data. AF and RC: analysis and interpretation of data. RC, YH, VO'F, and PL: critical revision. All authors contributed to the article and approved the submitted version.

## Conflict of Interest

The authors declare that the research was conducted in the absence of any commercial or financial relationships that could be construed as a potential conflict of interest.
