# Supplementary material for: Long Chain Fatty Acid Degradation Coupled to Biological Sulfidogenesis: A Prospect for Enhanced Metal Recovery
Source: Front Bioeng Biotechnol. 2020 Oct 23;8:550253. doi: 10.3389/fbioe.2020.550253 (PMC7644789; doi:10.3389/fbioe.2020.550253)

Long chain fatty acid degradation coupled to biological sulfidogenesis: a prospect for enhanced metal recovery

Anna Patrícya Florentino[1,*]; Rachel Biancalana Costa[2]; Yuansheng Hu[3]; Vincent O'Flaherty[1];

Piet N. L. Lens[1]

[1] Department of Microbiology, School of Natural Sciences and Ryan Institute, National University of Ireland Galway, University Road, Galway H91 TK33, Ireland

[2] Department of Biochemistry and Organic Chemistry, Institute of Chemistry, São Paulo State University, Araraquara, SP, Brazil.

[3] Department of Civil Engineering, School of Engineering, College of Science and Engineering, NUI Galway.

Figure S1 – Flow chart of the study

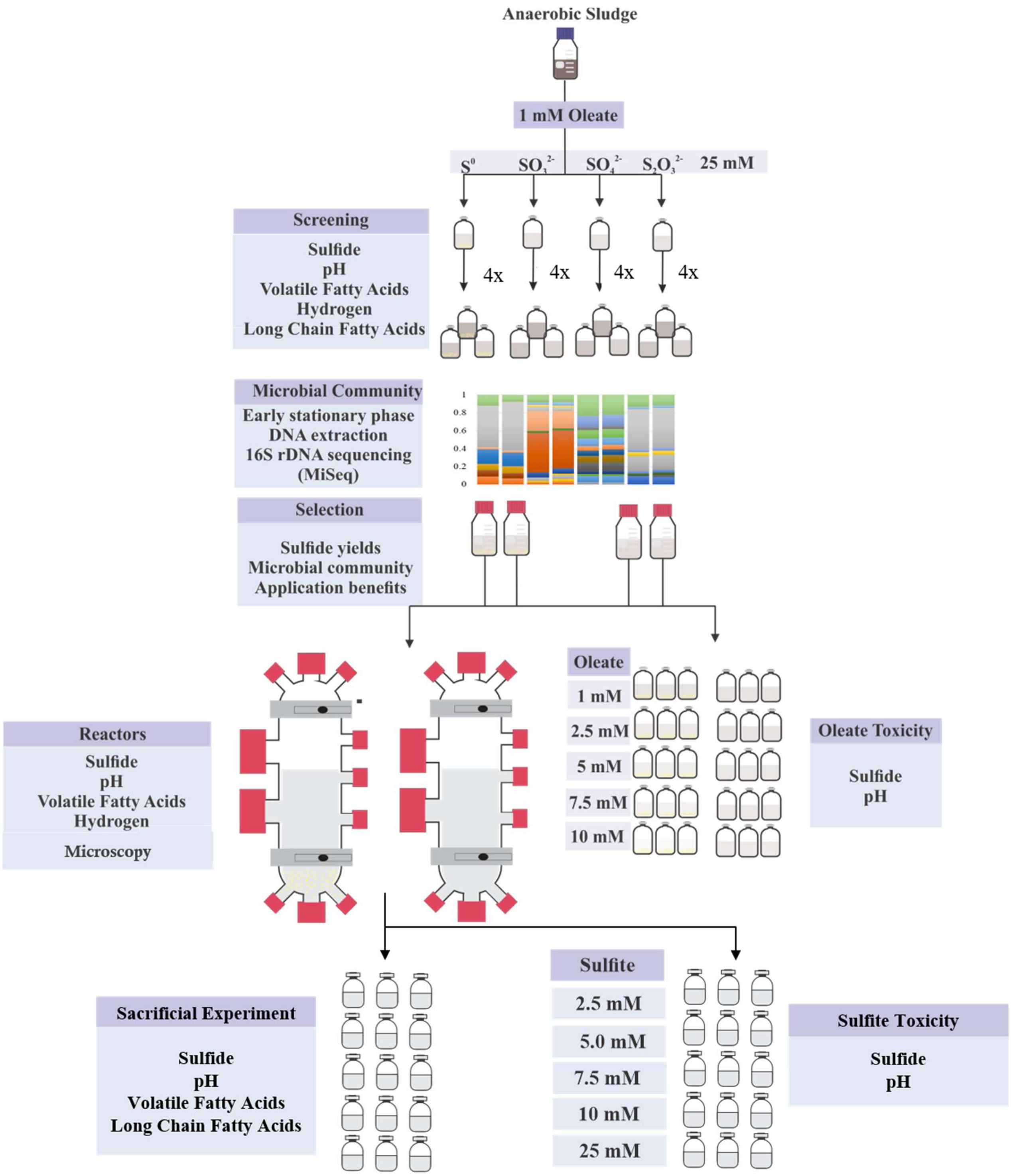

Supplement: Supplementary file 1 [file Image_1.pdf]
